# Supplementary material for: Regulatory Mechanisms of Epigenetic miRNA Relationships in Human Cancer and Potential as Therapeutic Targets
Source: Cancers (Basel). 2020 Oct 11;12(10):2922. doi: 10.3390/cancers12102922 (PMC7600069; doi:10.3390/cancers12102922)
Supplement: Supplementary file 1 [file cancers-12-02922-s001.pdf]

Regulatory Mechanisms of Epigenetic miRNA Relationships in Human Cancer and Potential as Therapeutic Targets

K.M. Taufiqul Arif, Esther K. Elliott, Larisa M. Haupt and Lyn R. Griffiths

Table S1. Epigenetic regulation of miRNAs in leukaemia and lymphomas.

Cancer/Condition	Epi Modification	Epi Regulator	miRNA	Level of Expression	Ref (Pubmed ID)
Acute Lymphoblastic leukaemia	DNA Methylation	DNMTs	miR-22	Low	19807731
	Histone Acetylation	HDACs	miR-141, miR-151a-5p, miR-15a, miR-181a-2, miR-181d, miR-185, miR-18a-3p, miR-192, miR-194-1, miR-196a-3p, miR-215, miR-26a-2-3p, miR-27a, miR-28-5p, miR-29a-5p, miR-29c, miR-320a, miR-339-3p, miR-377-5p, miR-491-3p, miR-500a, miR-502-3p, miR-509-3-5p, miR-550a-3p, miR-589, miR-769-5p, miR-887, miR-9-3p, miR-106b, miR-181c, miR-191, miR-24-2-5p, miR-29b-3p, miR-30c-2, miR-552, miR-574-3p, miR-923, miR-937, miR-205		
	H3K27me3/H3ac	-	miR-22	High	
	DNA Methylation	-	miR-9-1	Low	21810092
	DNA Methylation	DNMTs	miR-124-1, miR-124-2, miR-124-3, miR-34b,miR-34c, miR-9-1, miR-9-3, miR-10b, miR-203a, miR-196b, miR-9-2, miR-132, miR-212	Low	19164206
	H3K9me2/H3K4me3	-	miR-9-1, miR-9-2, miR-9-3, miR-10b, miR-34b, miR-34c, miR-124-1, miR-124-2, miR-124-3, miR-132, miR-196b, miR-203a, miR-212		
	DNA Methylation	DNMTs	miR-124-3p	High	19435910
	H3K9me3/H3K27me3/H3ac	-			
	DNA Methylation	-	miR-196b	Low	20494936
	DNA Methylation	DNMT-1/MLL	miR-486, miR-424, miR-148a, miR-101-3p	Low	21116279
			miR-200b, miR-200a, miR-429, miR-152,miR-10a, miR-503, miR-432	High	
Acute myeloid leukaemia	DNA Methylation	AML1/ETO protein	miR-193a	High	23223432
		DNMTs	miR-370	Low	22900969
			miR-193a	High	21399664
			miR-34b, miR-34c	High	19258499
			miR-124-3p	High	18451139
Aggressive B-cell lymphoma	H4ac/H3K27me3	MYC/HDAC3/EZH2	miR-29a	High	23079660
Burkitt lymphoma	DNA Methylation	-	miR-9-1	Low	20930934
	DNA Methylation	DNMTs	miR-129-2	High	23406679

Haematological malignancy	DNA Methylation	DNMTs	miR-124-1	High	21544199
	H3K4me3	-		Low	
	DNA Methylation	DNMTs	miR-203a	High	21323860
	DNA Methylation	DNMTs	miR-34a	High	20118199
Leukaemia	DNA Methylation	-	miR-124-3p	low	22541098
	DNA Methylation	DNMTs	miR-143	High	21706045
	DNA Methylation	DNMTs	miR-1202, miR-1228, miR-149, miR-1225, miR-339-3p, miR-210, miR-502-3p, miR-126, miR-150, miR-943, miR-1255a, miR-195, miR-493, miR-223	Low	21518471
			miR-638, miR-663a, miR-92b	High	
	DNA Methylation	DNMTs	miR-203a	High	18538733
	DNA Methylation	AML1-ETO oncoprotein/HDAC1/DNMTs/MeCP2	miR-223	High	17996649
	H3ac/H4ac				
Mantle cell lymphoma	H4ac	HDAC3/Myc	miR-15a	High	22002311
	H5ac	HDAC3/Myc	miR-16-1	High	22002312
Mixed lineage leukemia-rearranged acute leukemias	H3ac/H3K4me3	MLL fusion proteins	miR-17, miR-18a, miR-19a, miR-20a, miR-19b-1, miR-92a-1	High	20133587
NK-T cell lymphoma	DNA Methylation	DNMTs	miR-146a	High	21610143
T-cell leukaemia	H3K9me/H3K27me	PRC2	miR-31	High	22264793

Table S2. Epigenetic regulations of miRNAs in solid cancers.

Cancer/Condition	Epi Modification	Epi Regulator	miRNA	Level of Expression	Ref (pubmed ID)
Breast	DNA Methylation	DNMTs	miR-9-1	High	17948228
	DNA Methylation	DNMTs	miR-9-1, miR-124-3, miR-148a, miR-152, miR-663a	High	18314617
	DNA Methylation	-	miR-196a-2	Low	19567675
	DNA Methylation	DNMTs	miR-200c, miR-141	High	20682048
	DNA Methylation	-	let-7a-3	Low	20848182
	DNA Methylation	-	miR-375	Low	20978187
	H3K9me2	-			
	DNA Methylation	DNMTs	miR-335	High	21289068
	DNA Methylation	-	miR-195, miR-497	Low	21350001
	DNA Methylation	DNMTs	miR-125b-5p	High	21444677
	Histone Acetylation	HDACs	miR-200a	Low	21926171
	DNA Methylation	DNMTs	miR-34a, miR-125b-5p, miR-126, miR-155, miR-193b, miR-378a, miR-424, miR-494, miR-768	Low	22076154
	DNA Methylation	-	miR-200a, miR-200b, miR-429	Low	22231446

	DNA Methylation	DNMTs	miR-203a	High	22393463
	DNA Methylation	DNMT-1	miR-148a, miR-152	High	22935141
	DNA Methylation	-	miR-200a, miR-200b, miR-200c, miR-141, miR-429	Low	23112837
	DNA Methylation	-	miR-31, miR-130a, let-7a-3, let-7b, miR-155, miR-137, miR-34b, miR-34c	Low	23342147
	DNA Methylation	Mel-18/DNMT-1	miR-205	High	23474752
	DNA Methylation	Kindlin 2/DNMT-3	miR-200a, miR-200b, miR-429	Low	23483548
	DNA Methylation	-	miR-10b	High	23125021
Apoptosis resistant breast cancer	Histone Acetylation	HDACs	miR-1, miR-22, miR-139, miR-143, miR-144, miR-153, miR-155, miR-191-3p, miR-194-1, miR-215, miR-202-5p, miR-335, miR-486, miR-519c-5p, miR-544a, miR-559, miR-568, miR-620, miR-627, miR-638, miR-641, miR-888	Low	21971930
Breast tumour cell cycle progression	H3K4me3	JARID1B	let-7e	High	21969366
Breast tumour-initiating cells	DNA Methylation	sp1	miR-34c	Low	22074923
Breast cancer metastasis	DNA Methylation	-	miR-124-3p	Low	22085528
TNBC	DNA Methylation	DNMTs	miR-31	High	22289355
Bladder	DNA Methylation	-	miR-200a, miR-205	Low	20473948
	DNA Methylation	-	miR-34a, miR-126, miR-200b-5p, miR-516a-1, miR-551a, miR-744, miR-1226-5p, miR-24-1-5p, miR-149, miR-193a, miR-210, miR-503, miR-1227, miR-1229	Low	21138856
	DNA Methylation	DNMTs	miR-152, miR-212, miR-328, miR-1224-3p, miR-9-3, miR-9-1, miR-9-2, miR-34b	High	
	DNA Methylation	DNMTs	miR-9-2	Low	
	H3K9me3	-	miR-193a, miR-212, miR-149, miR-328, miR-1224	Low	
	DNA Methylation	DNMTs	miR-517a	Low	21479368
	DNA Methylation	-	miR-203a	Low	21836020
	DNA Methylation	DNMTs	miR-9-1, miR-9-3, miR-10b, miR-34b, miR-124-1, miR-124-2, miR-124-3, miR-137, miR-200b, miR-203a, miR-409, miR-675	High	23200812
Cervical	DNA Methylation	DNMTs	miR-124-3p	High	20579385
	DNA Methylation	-	miR-124-3p, miR-34b, miR-203a	Low	21461574
	DNA Methylation	-	miR-149, miR-203a, miR-375	High	23324622
Colon	DNA Methylation	DNMTs	miR-517b, miR-517a, miR-372, miR-519d, miR-520g	Low	21785829
			miR-373	High	
	DNA Methylation	-	miR-34a, miR-34b, miR-34c	Low	23243217
Colorectal	DNA Methylation	DNMTs	miR-342	High	18264139
	DNA Methylation	DNMTs	miR-124-3p, miR-127	Low	18519671
	DNA Methylation	DNMT-1/DNMT-3b	miR-34b, miR-34c	High	
	H3K4me3	-	miR-34b, miR-34c	Low	

	DNA Methylation	DNMTs	miR-9-1, miR-129-2, miR-137	High	19521961
	H3ac	-	miR-9-1, miR-129-2, miR-137	High	
	DNA Methylation	DNMTs	miR-137	High	20682795
	DNA Methylation	DNMTs	miR-15b, miR-96, miR-99a, miR-106a, miR-129-1, miR-135b, miR-146a, miR-148a, miR-181c, miR-219-1, miR-338	Low	21610744
			miR-34b, miR-34c	High	
	DNA Methylation	-	miR-34b	Low	21636702
	DNA Methylation	DNMTs	miR-345	High	21665895
	DNA Methylation	-	miR-140, miR-142, miR-220b, miR-338, miR-564, miR-663a, miR-939, miR-1234, miR-1180, miR-1203, miR-1224, miR-1225, miR-1226, miR-1227, miR-1228, miR-1229, miR-126, miR-1301, miR-149, miR-200b, miR-203a, miR-339, miR-33b, miR-566, miR-572, miR-596, miR-637, miR-661, miR-671, miR-886, miR-935, miR-937, miR-943, let-7a-3, miR-1306, miR-133a-2, miR-10a, miR-124-1, miR-124-3, miR-127, miR-129-2, miR-137, miR-152, miR-193a, miR-34b, miR-34c, miR-375, miR-9-1, miR-9-3, miR-410, miR-431, miR-433, miR-543, miR-675	Low	21698188
	DNA Methylation	DNMTs	miR-1237, miR-1247, miR-1826, miR-219-2, miR-24-1, miR-27b, miR-602, miR-663b, miR-941-1, miR-941-3	High	
	DNA Methylation	DNMTs	miR-136, miR-142, miR-146a, miR-146b, miR-190a, miR-195, miR-205, miR-211, miR-218-2, miR-337, miR-371a, miR-372, miR-373, miR-431, miR-432, miR-433, miR-486, miR-488, miR-497, miR-885, miR-9-2, miR-944	Low	21734013
			miR-10b, miR-1-1, miR-124-1, miR-124-2, miR-124-3, miR-127, miR-129-1, miR-129-2, miR-133a-2, miR-137, miR-152, miR-153-2, miR-155, miR-193a, miR-196a-1, miR-338, miR-34b, miR-34c, miR-548b, miR-596, miR-598, miR-708, miR-873, miR-876, miR-9-3	High	
	H3K4me3/H3K79me2	-	miR-200b, miR-200a, miR-429, miR-17, miR-18a, miR-19a, miR-20a, miR-19b-1, miR-92a-1, miR-124-1, miR-9-3	Low	
	Histone Acetylation	HDACs	miR-17, miR-18a, miR-19a, miR-20a, miR-19b-3p, miR-92a-3p, miR-106a, miR-18b, miR-20b	Low	22308110
	DNA Methylation	DNMTs	miR-1-1, miR-133a-2	High	22766685
	DNA Methylation	DNMTs	miR-149	High	22821729
			let-7e, miR-1184, miR-1201, miR-1205, miR-1228-5p, miR-1247, miR-1249, miR-125a-5p, miR-126-5p, miR-1260a, miR-1274a, miR-1274b, miR-1280, miR-1307, miR-130b-5p, miR-132, miR-148a, miR-149, miR-181a-2, miR-181c, miR-183-3p, miR-193a-5p, miR-21-3p, miR-210, miR-222-5p, miR-26b-3p, miR-27a-5p, miR-31-3p, miR-324-3p, miR-326, miR-331-3p, miR-34a, miR-361-3p, miR-362-3p, miR-365a-3p, miR-376a-1, miR-424, miR-425-3p, miR-449a, miR-454-5p, miR-486-5p, miR-500a, miR-501-3p, miR-502-3p, miR-576-5p, miR-625, miR-628-5p, miR-642a, miR-664a-5p, miR-720, miR-877, miR-92b, miR-93-3p, miR-95, miR-96	Low	

	DNA Methylation	DNMTs	miR-124-3p, miR-34b, miR-34c	High	22870149
	DNA Methylation	-	miR-34b, miR-34c, miR-9-1	Low	22989523
Endometrial	DNA Methylation	DNMTs	miR-129-2	High	19887623
	DNA Methylation	-	miR-9-1, miR-9-2, miR-9-3, miR-149	Low	21868754
	DNA Methylation	DNMT-1	miR-152	High	
	DNA Methylation	-	miR-34b	High	22052540
Epithelial ovarian	DNA Methylation	DNMTs	miR-127, miR-337, miR-432, miR-495, miR-376c, miR-376a-1, miR-376b, miR-377, miR-410, miR-520e, miR-519e, miR-519d, mir-516a-1, miR-518a-3p, miR-372, miR-448, miR-507, miR-424, miR-514a-3p, miR-15a, miR-184, miR-34a, miR-34b, miR-95, miR-182	Low	18458333
	Histone Acetylation	HDACs	miR-127, miR-337, miR-432, miR-495, miR-376c, miR-376a-1, miR-376b, miR-377, miR-410, miR-520e, miR-519e, miR-519d, mir-516a-1, miR-518a-3p, miR-372, miR-448, miR-507, miR-424, miR-514a-3p, miR-15a, miR-184, miR-34a, miR-34b, miR-95, miR-182		
Gastric	DNA Methylation	-	miR-124-1, miR-124-2, miR-124-3	Low	19165869
	DNA Methylation	DNMTs	miR-512-5p, miR-517b, miR-526b, miR-518b, miR-515-5p	Low	19503096
	Histone Acetylation	HDACs	miR-517b, miR-526b, miR-518b, miR-515-5p	Low	
	H3ac/H3K4me	HDACs	miR-512-5p	High	
	DNA Methylation	DNMTs	miR-211, miR-432, miR-495, miR-9-1, miR-9-2, miR-9-3		20080834
	DNA Methylation	DNMTs	miR-181c	High	
	DNA Methylation	DNMTs	miR-129-2	High	20331975
	DNA Methylation	DNMTs	miR-196b	High	20662076
	DNA Methylation	DNMTs	miR-142-5p, miR-193a, miR-195, miR-196a-5p, miR-34b, miR-34c, miR-375, miR-498, miR-512-3p, miR-512-5p, miR-515-3p, miR-515-5p, miR-516b-3p, miR-516b-5p, miR-517a, miR-517b, miR-517c, miR-518a-3p, miR-518b, miR-518c, miR-518d, miR-518e, miR-518f, miR-519b, miR-519c, miR-519d, miR-519e, miR-520a, miR-520b, miR-520c, miR-520d, miR-520e, miR-520f, miR-520g, miR-520h, miR-521, miR-522, miR-523, miR-524, miR-525, miR-526a, miR-526b, miR-519c-5p, miR-527, miR-602, miR-9-5p, miR-518c-5p, miR-518f-5p, miR-519e-5p, miR-520a-5p, miR-520d-5p, miR-524-5p, miR-526b-3p, miR-517-5p	Low	20924086
			miR-34b, miR-34c	High	
	DNA Methylation	DNMTs	miR-212	High	21053104
	DNA Methylation	-	miR-137	Low	21221794
	DNA Methylation	-	miR-124-3p	Low	21365509
	DNA Methylation	-	miR-10a, miR-34b, miR-34c, miR-196b	Low	21562367
	DNA Methylation	DNMTs	miR-10b	High	
	DNA Methylation	-	miR-34b, miR-34c, miR-124-3p	Low	21914401
	DNA Methylation	DNMTs	miR-139	High	21925125
	H3K9ac	HER2			
	DNA Methylation	DNMTs	miR-9-1, miR-9-2, miR-9-3	High	21931274

	DNA Methylation	DNMTs	miR-25-5p, miR-127-3p, miR-193a-5p, miR-378a, miR-409, miR-570, miR-767	Low	21960261
			miR-34b, miR-129-2-3p	High	
	Histone Acetylation	HDACs	miR-34b, miR-129-2-3p, miR-25-5p, miR-127-3p, miR-193a-5p, miR-378a, miR-409, miR-570, miR-767	Low	
	DNA Methylation	DNMT-1	miR-148a	High	22167392
	DNA Methylation	DNMTs	miR-155	Low	22426647
	DNA Methylation	DNMTs	miR-9-5p	Low	22906743
	H3ac/H3K4me	HDACs			
	DNA Methylation	DNMTs	miR-378a	High	23333942
Glioblastoma	DNA Methylation	DNMTs	miR-137	Low	18577219
	DNA Methylation	DNMTs	miR-211	High	23183822
Hepatocellular carcinoma	DNA Methylation	DNMTs	miR-7-5p, miR-27b, miR-196a-5p, miR-424, miR-496, miR-197, miR-101-3p	Low	18593903
	DNA Methylation	DNMT-1	miR-1	High	
	Histone Acetylation	HDACs	miR-1, miR-7-5p, miR-27b, miR-196a-5p, miR-424, miR-496, miR-197, miR-101-3p	Low	
	DNA Methylation	-	miR-409, miR-564, miR-410, miR-219-2, miR-24-1, miR-126, miR-369, miR-412, miR-339	Low	19843643
	DNA Methylation	DNMTs	miR-124-3p, miR-203a, miR-375	High	
	DNA Methylation	DNMTs	miR-125b-5p	High	21703189
	H3ac	HDAC4/SP1	miR-200a	High	21837748
	DNA Methylation	DNMTs	miR-191	High	21969817
	DNA Methylation	DNMTs	miR-520e	High	22105365
	DNA Methylation	RNAPII	miR-122	Low	22140464
	H3K9ac/H3K14ac	HDAC1/HDAC3/EP300	miR-224, miR-452	High	22459148
	DNA Methylation	-	miR-10a, miR-10b, miR-196b	Low	22976466
	DNA Methylation	DNMTs	miR-125b-5p<br>miR-125a-5p	High	23079745
	DNA Methylation	DNMTs	miR-512-3p, miR-517a, miR-518c, miR-519d, miR-520c-3p, miR-521, miR-517c	Low	3142219
	Histone Acetylation	HDACs			
	DNA Methylation	-	let-7b, miR-122, miR-149, miR-200b	Low	23229728
	DNA Methylation	DNMTs	miR-335, miR-101-2, miR-146b, miR-497	High	23229728
	DNA Methylation	DNMT-1	miR-9-2, miR-9-3, miR-124-1, miR-124-2, miR-124-3, miR-129-2, miR-596, miR-1247	High	23364900
	H3K27me3	-	miRNA biogenesis genes	High	23398123
	DNA Methylation	-	miR-124-3p, miR-203a	Low	23487440
Resistance of Hepatocellular carcinoma	DNA Methylation	DNMTs	miR-193a-3p	High	22117060

Liver cancer metastasis	H3K27me3	EZH2	miR-139-5p, miR-125b-5p, miR-200b, let-7c, miR-101-3p	Low	22370893
Lung cancer	DNA Methylation	DNMT-1/DNMT-3B	let-7a-3	High	17308078
	H3K27me3/H3K9me2/H3K9ac	EZH2/G9a/HDAC	miR-212	High	22110741
	DNA Methylation	-	miR-9-1, miR-34b, miR-34c, miR-9-3, miR-193a	Low	23156677
	DNA Methylation	-	miR-135b	Low	23695671
Non-small cell lung carcinoma	DNA Methylation	-	miR-34a	Low	19736307
	DNA Methylation	DNMTs	miR-200c	High	20696752
	DNA Methylation	-	miR-34b, miR-34c	Low	21383543
	DNA Methylation	DNMTs	miR-375, miR-196b, miR-127, miR-203a, miR-148a, miR-181c, miR-30e, miR-449a, miR-340, miR-486, miR-483, miR-150	Low	21702040
			miR-34b, miR-126	High	
	DNA Methylation	-	miR-152, miR-9-3, miR-124-1, miR-124-2, miR-124-3	Low	21917081
	DNA Methylation	DNMTs	miR-7-5p, miR-9-5p, miR-22, miR-29c, miR-30c-2, miR-33b, miR-34a, miR-92b, miR-99b, miR-125a, miR-127, miR-132, miR-140, miR-183, miR-193a, miR-194-1, miR-200c, miR-210, miR-375, miR-382, miR-423, miR-424, miR-432, miR-454, miR-455, miR-486, miR-487b, miR-494, miR-495, miR-720, miR-1180, miR-1203, miR-1323	Low	22282464
	DNA Methylation	DNMTs	miR-196a-5p	High	22876840
Small cell lung cancer	DNA Methylation	DNMTs	miR-34a, miR-34b, miR-34c	High	22047961
Melanoma	DNA Methylation	DNMTs	miR-34b, miR-489, miR-132, miR-142-3p, miR-200a, miR-145, miR-452, miR-21, miR-34c, miR-496, let-7e, miR-654, miR-519b	Low	21723283
			miR-375	High	
	Histone Acetylation	HDACs	miR-375, miR-34b, miR-489, miR-132, miR-142-3p, miR-200a, miR-145, miR-452, miR-21, miR-34c, miR-496, let-7e, miR-654, miR-519b	Low	
	DNA Methylation	DNMTs	miR-34b, miR-489, miR-375, miR-132, miR-142-3p, miR-200a, miR-145, miR-452, miR-21, miR-34c, miR-496, let-7e, miR-654, miR-519b	Low	21949788
			miR-34b	High	
	DNA Methylation	DNMTs	miR-376a-1	Low	22747855
	H3ac	HDACs	miR-376c	Low	
	H3ac	-	miR-376a-1	High	
	DNA Methylation	DNMTs	miR-212, miR-142-3p, miR-29b-1, miR-29b-2, miR-132-5p, miR-21, miR-222, miR-132, miR-21-3p, miR-100, miR-29b-1-5p, miR-29c, miR-30b-3p, miR-29a-5p, miR-155, miR-193a-5p, miR-194-1, miR-194-2, miR-26a-1, miR-26a-2, miR-331-3p, miR-363, miR-425, miR-215, miR-22, miR-30b, miR-140-3p, miR-190a, miR-224, miR-30c-1, miR-30c-2, miR-320a, miR-424, miR-99b	Low	22752337
	DNA Methylation	-	miR-34a, miR-602	Low	
	DNA Methylation	DNMTs	miR-182, miR-886-3p, miR-886-5p, miR-371a-5p, miR-146b-5p, miR-335, miR-424-3p, miR-412, miR-503	High	

	Histone Acetylation	HDACs	miR-182, miR-100, miR-132-5p, miR-140-3p, miR-142-3p, miR-146b-5p, miR-155, miR-190a, miR-193a-5p, miR-194-1, miR-194-2, miR-21-3p, miR-212, miR-215, miR-22, miR-222, miR-224, miR-26a-1, miR-26a-2, miR-29a-5p, miR-29b-1, miR-29b-1-5p, miR-29b-2, miR-29c, miR-30b-3p, miR-30c-1, miR-30c-2, miR-320a, miR-331-3p, miR-335, miR-363, miR-371a-5p, miR-412, miR-424-3p, miR-425, miR-485-3p, miR-503, miR-886-3p, miR-886-5p, miR-99b	Low	
	DNA Methylation	EZH2	miR-31	High	22948084
	DNA Methylation	DNMTs	miR-18b	High	23365201
Multiple myeloma	DNA Methylation	DNMTs	miR-203a	High	21707582
	DNA Methylation	DNMTs	miR-34b, miR-34c	High	21976676
Nasopharyngeal carcinoma	DNA Methylation	DNMTs	let-7a-1	Low	20440510
Neuroblastoma	DNA Methylation	-	miR-200b	Low	20574809
	DNA Methylation	-	miR-330, miR-27b, miR-23b, miR-24-1, miR-29b-2, miR-542, miR-381, miR-337, miR-154, miR-497, miR-191, miR-219-1, miR-196a-2, let-7i, miR-326, let-7f-1, let-7a-1, let-7a-3, miR-509-1, miR-509-3, miR-151a, miR-15b, miR-214, miR-10b, miR-639, miR-548a-3, miR-645, miR-181c, miR-218-1, miR-650, miR-218-2, miR-520d, miR-153-2, miR-153-1, miR-375, miR-7-3, miR-148a, miR-597	Low	22797059
	DNA Methylation	DNMTs	miR-140, miR-455, miR-29c, miR-126, miR-195, miR-10a, miR-425, miR-142, let-7e, miR-129-2, miR-26b, miR-101-1, miR-7a, miR-24-2, miR-203a, miR-1-1, miR-365b, miR-9-1, miR-641, miR-335, miR-184, miR-9-3, miR-296, miR-135a-1, miR-372, miR-149, miR-26a-2, miR-139, miR-340	High	
Oesophageal cancer	DNA Methylation	-	miR-375	Low	21533613
	DNA Methylation	DNMTs	miR-34a, miR-34b, miR-34c, miR-129-2	High	21547903
	DNA Methylation	DNMTs	miR-10a	Low	22966337
Oral cancer	DNA Methylation	DNMTs	miR-132, miR-34b, miR-137, miR-193a, miR-203a	High	18381414
	DNA Methylation	-	miR-375, miR-383, miR-483, miR-489, miR-500a	Low	21795477
	DNA Methylation	DNMTs	miR-218-5p, miR-585	High	
	DNA Methylation	DNMTs	miR-127, miR-137, miR-200a, miR-200b, miR-429, miR-200c, miR-141, miR-205	High	22132151
	DNA Methylation	-	miR-596		23233740
Osteosarcoma	H3ac	HDACs	miR-127, miR-411, miR-431, miR-432	High	22957032
Ovarian	DNA Methylation	DNMTs	let-7a-3	High	17974952
	DNA Methylation	-	miR-34a	Low	20145172
	DNA Methylation	DNMT-1	miR-199a-1, miR-125b-1	High	23146892
Multidrug resistance of ovarian cancer	DNA Methylation	DNMTs	miR-130b	High	22005523
Pancreatic cancer	DNA Methylation	DNMTs	miR-29a, miR-29b-3p, miR-103a-3p, miR-320a	Low	19407485
			miR-107	High	

	Histone Acetylation	HDACs	miR-107, miR-29a, miR-29b-3p, miR-103a-3p, miR-320a	Low	
	DNA Methylation	-	miR-148a	Low	20431052
	DNA Methylation	DNMTs	miR-200a, miR-200b	High	20551052
	DNA Methylation	DNMTs	miR-132	High	21665894
	DNA Methylation	DNMTs	miR-34a	Low	21909380
	Histone Acetylation	HDACs			
	DNA Methylation	DNMTs	miR-124-1, miR-124-2, miR-124-3	High	23334332
Prostate cancer	DNA Methylation	DNMTs	miR-126	High	19116145
	H3ac	HDAC2			
	DNA Methylation	DNMTs	miR-9-1, miR-9-2, miR-9-3, miR-27a, miR-32, miR-33a, miR-34a, miR-132, miR-149, miR-183, miR-188, miR-192, miR-193b, miR-194-1, miR-194-2, miR-203a, miR-215, miR-218-1, miR-218-2, miR-370, miR-375, miR-376a-1, miR-376a-2, miR-449a, miR-487b, miR-512-1, miR-512-2, miR-513a-1, miR-513a-2, miR-515-1, miR-515-2, miR-517a, miR-517b, miR-517c, miR-518b, miR-520f, miR-526a-1, miR-526a-2, miR-572, miR-601, miR-629, miR-630, miR-638, miR-663a, miR-765	Low	20073067
			miR-193b	High	
	Histone Acetylation	HDACs	miR-193b, miR-9-1, miR-9-2, miR-9-3, miR-27a, miR-32, miR-33a, miR-34a, miR-132, miR-149, miR-183, miR-188, miR-192, miR-193b, miR-194-1, miR-194-2, miR-203a, miR-215, miR-218-1, miR-218-2, miR-370, miR-375, miR-376a-1, miR-376a-2, miR-449a, miR-487b, miR-512-1, miR-512-2, miR-513a-1, miR-513a-2, miR-515-1, miR-515-2, miR-517a, miR-517b, miR-517c, miR-518b, miR-520f, miR-526a-1, miR-526a-2, miR-572, miR-601, miR-629, miR-630, miR-638, miR-663a, miR-765	Low	
	DNA Methylation	-	miR-145	Low	20588276
	DNA Methylation	DNMTs	miR-145	High	21349819
	DNA Methylation	DNMTs	miR-205	High	21368878
	DNA Methylation	DNMTs	let-7g-3p, let-7i-3p, miR-10a-3p, miR-106b-3p, miR-132, miR-146b-5p, miR-148a, miR-15b-3p, miR-152, miR-17, miR-17-3p, miR-18a, miR-18b, miR-19a, miR-19a-5p, miR-19b-3p, miR-19b-1-5p, miR-181c, miR-193b, miR-193b-5p, miR-199b-5p, miR-212, miR-219-1-3p, miR-20a-3p, miR-20b-3p, miR-203a, miR-22, miR-25-5p, miR-330-5p, miR-363, miR-375, miR-449b, miR-497, miR-483-5p, miR-503, miR-542-5p, miR-548b-5p, miR-550a-3p, miR-564, miR-615-5p, miR-616, miR-627, miR-636, miR-641, miR-642a, miR-9-3p, miR-935, miR-937, miR-92a-1-5p, miR-941	Low	22310291
			miR-132, miR-127, miR-146b-3p, miR-18a-3p, miR-330-3p, miR-34b, miR-34c, miR-376a-1, miR-376c, miR-409-3p, miR-410, miR-411, miR-432, miR-432-3p, miR-485-3p, miR-487b, miR-450a-5p, miR-539, miR-542-3p, miR-589, miR-655	High	
	DNA Methylation	DNMTs	miR-29a, miR-1256	High	22805767

	DNA Methylation	DNMTs	miR-205	High	22869146
	DNA Methylation	DNMTs	miR-124-3p	High	23069658
	DNA Methylation	DNMTs	miR-23b	High	23074286
	H3ac	-	miR-21	Low	20890304
	H3K4me3	HDAC1/HDAC2/HDAC4	miR-34b	Low	23147995
	DNA Methylation	-			
	DNA Methylation	-	miR-31	Low	23233736
Testicular cancer	DNA Methylation	-	miR-199a-5p	Low	21383689

© 2020 by the authors. Licensee MDPI, Basel, Switzerland. This article is an open access article distributed under the terms and conditions of the Creative Commons Attribution (CC BY) license (http://creativecommons.org/licenses/by/4.0/).
